# Mental Health and Related Factors after the Great East Japan Earthquake and Tsunami

**DOI:** 10.1371/journal.pone.0102497

**Published:** 2014-07-24

**Authors:** Yukari Yokoyama, Kotaro Otsuka, Norito Kawakami, Seiichiro Kobayashi, Akira Ogawa, Kozo Tannno, Toshiyuki Onoda, Yumi Yaegashi, Kiyomi Sakata

**Affiliations:** 1 Department of Hygiene and Preventive Medicine, School of Medicine, Iwate Medical University, Iwate, Japan; 2 Department of Social Welfare, Nihon Fukushi University, Aichi, Japan; 3 Department of Psychiatry and Disaster Medicine, School of Medicine, Iwate Medical University, Iwate, Japan; 4 Department of Mental Health, Graduate School of Medicine, The University of Tokyo, Tokyo, Japan; 5 Department of Plastic and Reconstructive Surgery, School of Medicine, Iwate Medical University, Iwate, Japan; 6 Iwate Medical University, Iwate, Japan; Columbia University Medical Center, United States of America

## Abstract

Mental health is one of the most important issues facing disaster survivors. The purpose of this study is to determine the prevalence and correlates of mental health problems in survivors of the Great East Japan Earthquake and Tsunami at 6–11 months after the disaster. The questionnaire and notification were sent to the survivors in three municipalities in the Tohoku area of the Northern part of Honshu, Japan’s largest island, between September 2011 and February 2012. Questionnaires were sent to 12,772, 11,411, and 18,648 residents in the Yamada, Otsuchi, and Rikuzentakata municipalities, respectively. Residents were asked to bring the completed questionnaires to their health check-ups. A total of 11,124 or (26.0%) of them underwent health check-ups, and 10,198 were enrolled. We excluded 179 for whom a K6 score was missing and two who were both 17 years of age, which left 10,025 study participants (3,934 male and 6,091 female, mean age 61.0 years). K6 was used to measure mental health problems. The respondents were classified into moderate (5–12 of K6) and serious mental health problems (13+). A total of 42.6% of the respondents had moderate or serious mental health problems. Multivariate analysis showed that women were significantly associated with mental health problems. Other variables associated with mental health problems were: younger male, health complaints, severe economic status, relocations, and lack of a social network. An interaction effect of sex and economic status on severe mental health problems was statistically significant. Our findings suggest that mental health problems were prevalent in survivors of the Great East Japan Earthquake and Tsunami. For men and women, health complaints, severe economic status, relocations, and lack of social network may be important risk factors of poor mental health. For men, interventions focusing on economic support may be particularly useful in reducing mental health problems after the disaster.

## Introduction

On March 11, 2011, Japan was rocked by a magnitude 9.0 earthquake that caused serious damages to the Tohoku area in the northern part of the main island of Japan. The tsunami that followed the earthquake devastated the coastal areas, and as of November 2012, 15,873 people have been confirmed dead, and 2,768 are still missing [1]. The widespread, costly damage has been referred to as the worst natural disaster in the recorded history of Japan.

Mental health is one of the most important issues for disaster survivors, and many studies have reported higher rates of mental health problems after disasters (e.g., hurricanes and tsunamis) [2]–[5]. In Japan, mental health problems have been a matter of great concern after disasters, such as the Hanshin Awaji Earthquake in 1995 that killed more than 5,500 people [6] and the Niigata-Chuetsu Earthquake in 2004 that forced the evacuation of 103, 000 residents [7]. However, the Great East Japan Earthquake wreaked enormous damage, surpassing that of previous disasters. Previous research led us to expect a high prevalence of mental health problem among the victims of the Great East Japan Earthquake.

It is essential to identify vulnerable populations and give them effective assistance. Available evidence shows that risk factors for mental health include age [8], female sex [9]–[11], low socioeconomic status [9], relocation [12], and lack of social network [8], [9].

However, no previous studies allowed us to clarify the factors associated with mental health in the large population of the disaster-stricken area after the 2011 Great East Japan Earthquake.

In addition, despite the consistent sex differences observed in the prevalence of mental health problems after disasters [9]–[11], previous studies have not sufficiently established specific risk factors by sex. Females are more likely to have mental health problems, regardless of whether assessments occurred in the aftermath of a disaster [13], [14]. However, risk factors, such as unemployment are known to have more serious repercussions on mental health among males [15]. Previous studies conducted in Japan have revealed that increasing opportunities for social participation improves mental health, especially for females [16]. Therefore, determining sex specific stressors could provide important insights into the appropriate interventions needed for facilitating coping, healing, and recovery from loss and catastrophe. The primary objective of the present study was to report the prevalence of mental health problems among survivors of the 2011 Great East Japan Earthquake and Tsunami. The secondary objective was to examine the factors associated with mental health according to sex.

## Materials and Methods

### Study populations

We analyzed part of data from the RIAS (RIAS; Research project for prospective Investigation of health problems Among Survivors of the Great East Japan Earthquake and Tsunami Disaster). It was launched with the purpose of screening the physical, mental, and social health of residents in the devastated area, and determining the long-term health impact of the disaster. The survey was carried out between September 2011 and February 2012 in 3 municipalities in Iwate Prefecture located in the Tohoku area in the northern part of Honshu, Japan’s largest island. The municipalities were Yamada Town, Otsuchi Town and Rikuzentakata City, which were heavily damaged by the earthquake, and broad areas were washed away by the tsunami. Seven hundred and ninety-nine people died or were missing in Yamada, 1,811 in Rikuzentakata, and 1,311 in Otsuchi [17], which accounted for 4.3%, 7.8%, and 8.6% of the total population in each municipality respectively. Otsuchi sustained the second highest ratio of dead and missing in Japan, Rikuzentakata the third, and Yamada the fifth.

Although at that time we could not know for certain the population remaining after the disaster (owing to various factors such as residents dying, going missing, or relocating), we sent out notifications of the health survey and questionnaires to all residents aged 18 years or older based on provisional figures compiled by municipalities: 12,772 people in Yamada, 11,411 in Otsuchi, and 18,648 in Rikuzentakata. Many survivors had informed their municipal government of their addresses when filing their claims for accident compensation. As some residents did not live at the addresses captured by the government’s system, we announced our health check-ups on a community bulletin board located at the temporary housing complexes in addition to mailing notifications and questionnaires to residents whose addresses were registered with their municipal government. Most of the relocated survivors lived in the same areas compensation filing system where their temporary housing complexes were situated, and had received information about the check-ups from the municipality. Thus, there is a high possibility that residents of the devastated areas were well aware of the health checks. We asked residents to complete the questionnaire and bring it to their municipal check-up site. When the residents received a health check-up, we explained the study in detail. If the answers in the questionnaires were insufficient, a trained interviewer asked the respondent to answer as fully as possible.

**Figure 1:**
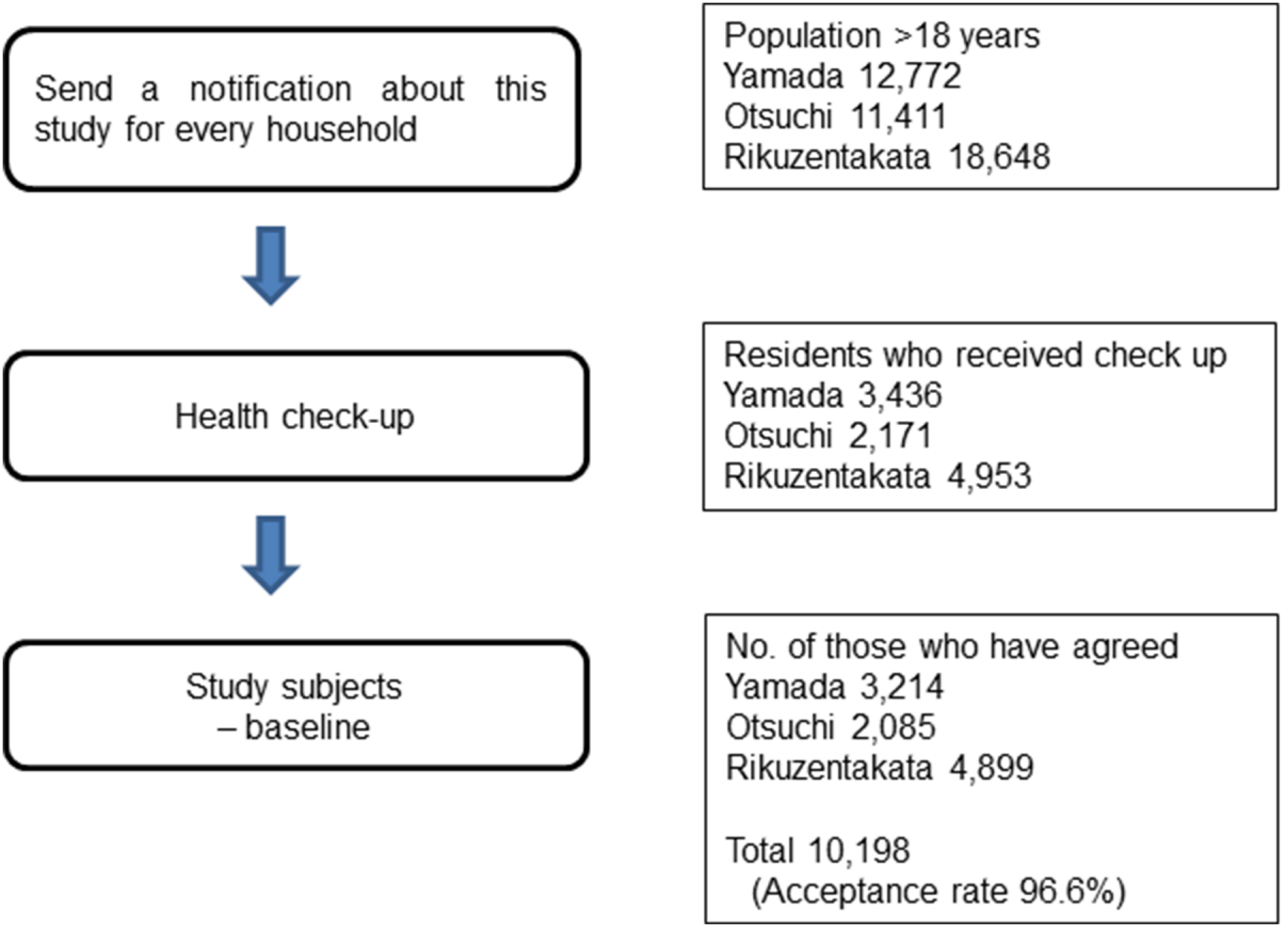
Study Design, Sample Selection, and Description of the Study Population.

Figure 1 shows the selection of the participants in this study. A total of 11,124 people underwent health check-ups, and 10,198 people gave written informed consent for participation in this study (acceptance rate: 91.7%). Participants comprised about 25% of the area population in the same age group in the 3 municipalities (See Appendix S1, S2, S3 and S4 for more information). Of these 10,198 participants, we excluded 179 for whom the mental health score was missing and 2 who were 17 years old. Consequently, 10,025 participants (3,934 male and 6,091 female) took part in the present study. Mean age (SD) was 62.3 (14.5) years and 60.1 (14.7) years respectively.

The study protocol was reviewed and approved by the Ethics Committee of Iwate Medical University.

### Measurements

#### Mental health

Mental health was assessed using the K6 scale [18], [19], which consisted of 6 items measuring mental health on a 5-point Likert scale. Each question was scored from 0 to 4, and the total points score therefore ranged from 0 to 24. In this study, Cronbach’s α coefficients were 0.88. Based on previous research on K6 in Japan, the respondents were classified into having a serious mental health problem (SMHP) (the score of 13+), moderate mental health problem (MMHP) (5–12), and no mental health problem (0–4) [3], [20]–[22].

#### Correlates

We collected by self-administered questionnaire information on demographic characteristics, presence or absence of health complaints, economic status, frequency of relocation, and social network. To assess health complaints, we asked respondents, “In the past few days, have you had any health problems?” (yes or no) Economic status was assessed by asking, “How do you feel about your current economic situation?’’ with 4 options: very severe, severe, slightly severe, and normal. On the basis of their answers, participants were categorized into 2 groups: severe (very severe, severe, and slightly severe) or normal. After the disaster, 386 evacuation centers were located in the Iwate Prefecture [23]. We asked them, “How many times have you been moved from an evacuation center?” with 5 options, no relocation, 1, 2, 3, and ≥4. Frequency of relocation was categorized into 3 groups: no relocation, 1 or 2, and ≥3. The Lubben’s Social Network Scale was used to assess social networks size [24], [25]. This measure used 6 questions: 3 questions evaluated 3 different aspects of social networks that are attributable to family ties and a parallel set to friendship ties. Although the original scale contained a definition sentence of family, we decided to avoid the description, taking into consideration the feelings of the bereaved. Each question was scored from 0 to 5. The total score ranged from 0 to 30, and a high score indicated a larger social network size. In this study, Cronbach’s α coefficients were 0.86. In previous studies, a cut-off point of <12 has been used to screen for social isolation. We classified respondents with scores of <12 as lacking a social network [24].

### Statistical Analysis

Participants were divided into five categories according to their age at survey: 18–44, 45–54, 55–64, 65–74, and ≥75 years. First, we presented characteristics of total, male and female participants by age categories and the chi-square test was used to evaluate differences in characteristics. Second, we also presented characteristics of total, male and female participants by K6 groups. Third, we examined the associations between K6 score and the following variables: sex, age, health complaints, economic status, frequency of relocations, and social network. Multinomial logistic regression was used to determine correlates of MMHPs (a score of 5–12 on the K6) and SMHPs (a score of 13+) relative to no mental health problem (0–4). In the multivariate models, the above variables and municipality were all adjusted for each other. Tests for an interaction between sex and each study variable were conducted by entering an interaction term for sex and each risk factor in multivariate models. All statistical analyses were performed with SPSS version 20.0 (IBM), and all statistical tests were 2-sided. P values <0.05 were considered to indicate statistical significance.

## Results

The characteristics are presented in Table 1. A total of 42.6% of the study population were classified with either MMHPs or SMHPs: 36.4% (31.2% of male participants, 39.7% of female participants) with MMHPs and 6.2% (4.5% of male participants, 7.3% of female participants) with SMHPs. The proportion of respondents with health complaints was about 50% in participants aged 18–44 years and 45–54 years, whereas in those aged 65–74 years, it was about 40%. In participants aged 55–65 years, 65–74 years, and 75 years older, about half of them experienced relocation from their former homes, whereas about 60% had relocated. Nearly half of the respondents in participants aged 18–44 years and 45–54 years had lacked a social network (score <12). The tendencies above were not much different between sexes.

**Table 1:** Demographic characteristics of the sample, 2011 (n = 10,025).

			Age Range, y												
		All	18–44		45–54		55–64			65–74			75 or older		
			(n = 1,650)		(n = 1,161)		(n = 2,520)			(n = 3,019)			(n = 1,675)		
		No. (%)	No. (%)		No. (%)		No. (%)			No. (%)			No. (%)		p value
**Total**															
Sex	Male	3934 (39.2)	583 (35.3)		404 (34.8)		913 (36.2)			1277 (42.3)			757 (45.2)		P<0.001
	Female	6091 (60.8)	1067 (64.7)		757 (65.2)		1607 (63.8)			1742 (57.7)			918 (54.8)		
Health complaint	Yes	4505 (45.6)	827 (50.6)		570 (49.8)		1151 (46.3)			1195 (40.3)			762 (46.4)		P<0.001
Economic status	Severe	5142 (51.4)	998 (60.5)		725 (62.5)		1408 (55.9)			1373 (45.7)			638 (38.2)		P<0.001
Relocation	No	4829 (48.4)	676 (41.0)		523 (45.2)		1248 (49.7)			1523 (50.8)			859 (51.8)		P<0.001
	1–2	2972 (29.8)	563 (34.2)		366 (31.6)		749 (29.8)			844 (28.2)			450 (27.1)		
	≥3	2172 (21.8)	409 (24.8)		269 (23.2)		515 (20.5)			630 (21.0)			349 (21.0)		
Social network	<12	4084 (41.6)	753 (46.1)		541 (47.5)		1065 (43.0)			1089 (36.9)			636 (39.2)		P<0.001
K6	0–4	5753 (57.4)	892 (54.1)		603 (51.9)		1358 (53.9)			1858 (61.5)			1042 (62.2)		P<0.001
	5–12	3648 (36.4)	622 (37.7)		475 (40.9)		1014 (40.2)			1005 (33.3)			532 (31.8)		
	13–24	624 (6.2)	136 (8.2)		83 (7.1)		148 (5.9)			156 (5.2)			101 (6.0)		
				**Age Range, y**											
		**All**		**18–44**		**45–54**	**55–64**		**65–74**			**75 or older**			
		**No. (%)**		**No. (%)**		**No. (%)**	**No. (%)**		**No. (%)**			**No. (%)**		**p value**	
**Male participants**															
Health complaint	Yes	1534 (39.5)		259 (44.8)		169 (42.3)	344 (38.2)		440 (35.0)			322 (43.3)		P<0.001	
Economic status	Severe	2082 (53.1)		386 (66.3)		273 (67.7)	539 (59.1)		592 (46.7)			292 (38.6)		P<0.001	
Relocation	No	1943 (49.7)		243 (41.8)		180 (44.8)	448 (49.3)		662 (52.2)			410 (54.7)		P<0.001	
	1–2	1177 (30.1)		222 (38.1)		133 (33.1)	279 (30.7)		358 (28.2)			185 (24.7)			
	≥3	791 (20.2)		117 (20.1)		89 (22.1)	182 (20.0)		248 (19.6)			155 (20.7)			
Social network	<12	1660 (43.0)		293 (50.8)		197 (49.4)	395 (43.9)		467 (37.3)			308 (42.1)		P<0.001	
K6	0–4	2528 (64.3)		320 (54.9)		239 (59.2)	563 (61.7)		882 (69.1)			524 (69.2)		P<0.001	
	5–12	1228 (31.2)		215 (36.9)		143 (35.4)	304 (33.3)		359 (28.1)			207 (27.3)			
	13–24	178 (4.5)		48 (8.2)		22 (5.4)	46 (5.0)		36 (2.8)			26 (3.4)			
**Female participants**															
Health complaint	Yes	2971 (49.5)		568 (53.7)		401 (53.8)	807 (50.9)	755 (44.1)			440 (49.0)			P<0.001	
Economic status	Severe	3060 (50.4)		612 (57.4)		452 (59.7)	869 (54.1)	781 (45.1)			346 (37.9)			P<0.001	
Relocation	No	2886 (47.6)		433 (40.6)		343 (45.4)	448 (49.3)	861 (49.8)			449 (49.9)			P<0.001	
	1–2	1795 (29.6)		341 (32.0)		233 (30.8)	279 (30.7)	486 (28.1)			265 (29.2)				
	≥3	1381 (22.8)		292 (27.4)		180 (23.8)	182 (20.0)	382 (22.1)			194 (21.4)				
Social network	<12	2424 (40.6)		460 (43.6)		344 (46.4)	670 (42.5)	622 (36.5)			328 (36.9)			P<0.001	
K6	0–4	3225 (52.9)		572 (53.6)		364 (48.1)	795 (49.5)	976 (56.0)			518 (56.4)			P<0.001	
	5–12	2420 (39.7)		407 (38.1)		332 (43.9)	710 (44.2)	646 (37.1)			325 (35.4)				
	13–24	446 (7.3)		88 (8.2)		61 (8.1)	102 (6.3)	120 (6.9)			75 (8.2)				

Numbers vary because of missing data, perceived symptom; male (55) female (91), economic status; male (12) female (15), relocation; male (23) female (29), and social network; male (76) female (125).

No case (K6; 0–4), MMHP, mild mental health problem (K6; 5–12), SMHP, severe mental health problem (K6; 13–24).

Table 2 shows the descriptive data according to K6 score. Female participants were more likely to have MMHPs and SMHPs than male participants. More than 70% of respondents with SMHPs reported having health complaints, and severe economic status. These were also reported by about 60% of those with MMHPs. Nearly one third of those with SMHPs reported ≥3 relocations, whereas one quarter of MMHPs reported the same. Separated by sex, more male (68.0% of MMHPs, 83.1% of SMHPs) than female (61.6%, 71.5%) respondents had reported severe economic status. In male, 60% of respondents with SMHPs and 47.6% of those with MMHPs lacked a social network, whereas in female, 53.1% of SMHPs and 44% of MMHPs did.

**Table 2:** Characteristics according to K6 score (n = 10,025).

		K6			
		no case	MMHP	SMHP	
		(n = 5,753)	(n = 3,648)	(n = 624)	
		No. (%)	No. (%)	No. (%)	p
**Total**					
Sex	Male	2528 (43.9)	1228 (33.7)	178 (28.5)	P<0.001
Age (yr)	18–44	892 (15.5)	622 (17.1)	136 (21.8)	P<0.001
	45–54	603 (10.5)	475 (13.0)	83 (13.3)	
	55–64	1358 (23.6)	1014 (27.8)	148 (23.7)	
	65–74	1858 (32.3)	1005 (27.5)	156 (25.0)	
	≥75	1042 (18.1)	532 (14.6)	101 (16.2)	
Health complaint	Yes	2005 (35.3)	2040 (56.9)	460 (75.3)	P<0.001
Economic status	Severe	2355 (41.1)	2321 (63.7)	466 (74.8)	P<0.001
Relocation	0	3027 (53.0)	1575 (43.3)	227 (36.5)	
	2-Jan	1619 (28.3)	1152 (31.7)	201 (32.3)	
	≥3	1070 (18.7)	908 (25.0)	194 (31.2)	
Social network	<12	2132 (37.8)	1614 (45.2)	338 (55.2)	P<0.001
**Male participants**					
Age (yr)	18–44	320 (12.7)	215 (17.5)	48 (27.0)	P<0.001
	45–54	239 (9.5)	143 (11.6)	22 (12.4)	
	55–64	563 (22.3)	304 (24.8)	46 (25.8)	
	65–74	882 (34.9)	359 (29.2)	36 (20.2)	
	≥75	524 (20.7)	207 (16.9)	26 (14.6)	
Health complaint	Yes	792 (31.7)	615 (51.1)	127 (71.8)	P<0.001
Economic status	Severe	1100 (43.7)	834 (68.0)	148 (83.1)	P<0.001
Relocation	0	1342 (53.5)	531 (43.3)	70 (39.5)	P<0.001
	2-Jan	711 (28.3)	408 (33.3)	58 (32.8)	
	≥3	455 (18.1)	287 (23.4)	49 (27.7)	
Social network	<12	980 (39.6)	574 (47.6)	106 (60.6)	P<0.001
**Female participants**					
Age (yr)	18–44	572 (17.7)	407 (16.8)	88 (19.7)	P<0.001
	45–54	364 (11.3)	332 (13.7)	61 (13.7)	
	55–64	795 (24.7)	710 (29.3)	102 (22.9)	
	65–74	976 (30.3)	646 (26.7)	120 (26.9)	
	≥75	518 (16.1)	325 (13.4)	75 (16.8)	
Health complaint	Yes	1213 (38.1)	1425 (59.7)	333 (76.7)	P<0.001
Economic status	Severe	1255 (39.0)	1487 (61.6)	318 (71.5)	P<0.001
Relocation	0	1685 (52.5)	1044 (43.3)	157 (35.3)	P<0.001
	2-Jan	908 (28.3)	744 (30.9)	143 (32.1)	
	≥3	615 (19.2)	621 (25.8)	145 (32.6)	
Social network	<12	1152 (36.4)	1040 (44.0)	232 (53.1)	P<0.001

Numbers vary because of missing data, perceived symptom; male (55) female (91), economic status; male (12) female (15), relocation; male (23) female (29), and social network; male (76) female (125). No case (K6; 0–4), MMHP, mild mental health problem (K6; 5–12), SMHP, severe mental health problem (K6; 13–24).

Table 3 shows the results of multinomial logistic regression analysis with the no case group as the reference category. Multivariate analysis showed that female respondents were more likely to have MMHPs [odds ratio (OR), 1.5; 95% confidence interval (CI), 1.4–1.7] and SMHPs (OR, 1.8; 95% CI, 1.5–2.3). In multivariate models, the factors associated with both MMHPs and SMHPs were: health complaints (OR, 2.1; 95% CI, 1.9–2.3 and OR, 4.6; CI, 3.8–5.6); severe economic status (OR, 2.3; 95% CI, 2.1–2.5 and OR, 3.4; 95% CI, 2.8–4.2); 1 to 2 relocations (OR, 1.2; 95% CI, 1.1–1.4 and OR, 1.4; 95% CI, 1.2–1.8); ≥3 relocations (OR, 1.4; 95% CI, 1.3–1.6 and OR, 2.0; 95% CI, 1.6–2.4); and lack of social network (OR, 1.3; 95% CI, 1.1–1.4 and OR, 1.8; 95% CI, 1.5–2.1).

**Table 3:** Mutinomial logistic regressions for the categories of mental health.

	MMHP				SMHP					
	COR	95% CI	AOR	95% CI	COR	95% CI	AOR	95% CI		
Sex										
Female	1.55	(1.42–1.68)	1.52	(1.39–1.67)	1.96	(1.64–2.36)	1.87	(1.54–2.26)		
Male	1.00		1.00		1.00		1.00			
Age (yr)										
<44	1.29	(1.13–1.47)	1.02	(0.89–1.17)	1.82	(1.42–2.32)	1.24	(0.96–1.61)		
45–54	1.46	(1.26–1.68)	1.13	(0.97–1.31)	1.64	(1.24–2.17)	1.06	(0.78–1.43)		
55–64	1.38	(1.23–1.54)	1.18	(1.04–1.33)	1.30	(1.03–1.64)	1.01	(0.78–1.29)		
65–74	1.00		1.00		1.00		1.00			
≥75	0.94	(0.83–1.07)	0.98	(0.85–1.13)	1.15	(0.89–1.50)	1.19	(0.90–1.58)		
Health complaint										
Yes	2.42	(2.22–2.63)	2.13	(1.95–2.33)	5.58	(4.61–6.76)	4.57	(3.75–5.57)		
No	1.00		1.00		1.00		1.00			
Economic status										
Severe	2.52	(2.32–2.75)	2.26	(2.06–2.48)	4.26	(3.53–5.14)	3.44	(2.82–4.20)		
Normal	1.00		1.00		1.00		1.00			
Relocation										
≥3	1.63	(1.46–1.82)	1.40	(1.25–1.58)	2.42	(1.97–2.96)	1.95	(1.57–2.43)		
1 or 2	1.37	(1.24–1.51)	1.23	(1.11–1.37)	1.66	(1.36–2.02)	1.43	(1.16–1.77)		
no	1.00		1.00		1.00		1.00			
Social network										
<12	1.36	(1.25–1.48)	1.25	(1.14–1.37)	2.03	(1.72–2.40)	1.75	(1.47–2.09)		
≥12	1.00		1.00		1.00		1.00			

Abbreviations: COR, crude odds ration; AOR, adjusted odds ratio; CI, confidential interval; MMHP, mild mental health problem; SMHP, severe mental health problem. OR are calculated by using a multinomial logistic regression model with no case (K6; 0–4) as a reference group. AOR were adjusted for the effect of all other variables shown in the table and municipality.

The results of bivariate and multivariate analyses showed similar tendencies, except with regard to the age category. In bivariate analysis, younger or middle-aged survivors were more likely to have mental health problems. However, this association was attenuated in the multivariate model. Table 4 shows the association between mental health and independent variables by sex. In both sexes, MMHPs and SMHPs were more frequent in those with health complaints, severe economic status, and limited social network, after adjustment for all other variables. Sex differences were observed for SMHPs. Multivariate analysis showed that men aged <44 years (OR, 2.4; 95% CI, 1.5–3.9) were more likely to have had SMHPs as compared with those aged 65–74 years, while no such trend was observed among female participants. An interaction effect of sex and age on SMHP was statistically significant (P<0.01). In addition, men with severe economic hardships showed a higher risk of developing SMHPs (OR, 4.9; 95% CI, 3.2–7.5) compared with women (OR, 3.0; 95% CI, 2.4–3.8). An interaction effect of sex and economic status on SMHP was statistically significant. For women, experiences of relocation were associated with SMHPs, whereas no such association was found for men. However, apart from age and economic status, the interaction between sex and other study variables was not statistically significant.

**Table 4:** Multivariate multinomial regression of mental health by sex.

		MMHP				SMHP							
		COR	95% CI	AOR	95% CI	COR	95% CI			AOR	95% CI		
**Male**													
Age (yr)	<44	1.65	(1.34–2.04)	1.29	(1.03–1.61)	3.67	(2.34–5.77)			2.44	(1.52–3.92)		
	45–54	1.47	(1.16–1.87)	1.15	(0.89–1.49)	2.26	(1.30–3.91)			1.48	(0.84–2.63)		
	55–64	1.33	(1.10–1.60)	1.14	(0.93–1.39)	2.00	(1.28–3.14)			1.50	(0.94–2.41)		
	≥75	0.97	(0.79–1.19)	0.97	(0.78–1.21)	1.22	(0.73–2.04)			1.23	(0.72–2.12)		
Health complaint	Yes	2.25	(1.96–2.60)	2.08	(1.79–2.41)	5.47	(3.90–7.68)			4.63	(3.27–6.55)		
Economic status	Severe	2.73	(2.37–3.16)	2.42	(2.08–2.82)	6.36	(4.26–9.48)			4.93	(3.24–7.49)		
Relocation	≥3	1.59	(1.33–1.91)	1.29	(1.07–1.57)	2.06	(1.41–3.02)			1.37	(0.91–2.05)		
	1 or 2	1.45	(1.24–1.70)	1.23	(1.04–1.45)	1.56	(1.09–2.24)			1.14	(0.78–1.67)		
Social network	<12	1.39	(1.21–1.59)	1.27	(1.10–1.47)	2.35	(1.71–3.21)			1.94	(1.40–2.69)		
**Female**													
Age (yr)	<44	1.08	(0.91–1.26)	0.88	(0.74–1.05)	1.25	(0.93–1.68)			0.90	(0.66–1.23)		
	45–54	1.38	(1.15–1.65)	1.11	(0.91–1.34)	1.36	(0.98–1.90)			0.92	(0.65–1.32)		
	55–64	1.35	(1.17–1.55)	1.19	(1.02–1.39)	1.04	(0.79–1.38)			0.86	(0.64–1.16)		
	≥75	0.95	(0.80–1.12)	1.00	(0.83–1.20)	1.18	(0.87–1.60)			1.22	(0.87–1.70)		
Health complaint	Yes	2.41	(2.16–2.68)	2.17	(1.94–2.43)	5.35	(4.23–6.76)			4.62	(3.63–5.89)		
Economic status	Severe	2.51	(2.25–2.79)	2.16	(1.93–2.43)	3.91	(3.15–4.87)			2.99	(2.37–3.76)		
Relocation	≥3	1.63	(1.42–1.87)	1.49	(1.28–1.72)	2.53	(1.98–3.23)			2.31	(1.78–3.01)		
	1 or 2	1.32	(1.17–1.50)	1.23	(1.08–1.40)	1.69	(1.33–2.15)			1.57	(1.21–2.03)		
Social network	<12	1.37	(1.23–1.53)	1.23	(1.10–1.38)	1.98	(1.62–2.42)			1.68	(1.35–2.07)		

Abbreviations: COR, crude odds ration; AOR, adjusted odds ratio; CI, confidential interval; MMHP, mild mental health problem; SMHP, severe mental health problem. OR are calculated by using a multinomial logistic regression model with no case (K6; 0–4) as a reference group. Adjusted OR were adjusted for the effect of all other variables shown in the table and municipality.

## Discussion

The present study revealed that mental health problems were prevalent among survivors of the 2011 earthquake and tsunami in Japan, at 6–11 months after the disaster. We identified female sex, younger male, having health complaints, severe economic status relocations, and lack of social networks were associated with having moderate or serious mental health problem. In sex-stratified analysis, younger males and males with severe economic status were more likely to have severe mental health problem than their counterparts among females.

Consistent with other evidence of the negative effects of disasters on mental health [2]–[5], our results suggested that the Great-East earthquake lead to a host of problems in the residents of the disaster-stricken area. In our study, 42.6% of survivors were determined to have MMHPs (a K6 score ranging from 5 to 12) or SMHPs (a K6 score of 13–24). A previous study which was conducted among a representative community-based sample of residents in Japan aged 20–74 showed that 26.8% in respondents had a K6 score of 5 or higher [21]. Another study conducted in Japanese aged 25–59 showed 30.6% with the same scoring procedure [14]. The prevalence of SMHPs (6.2%) in the present study was higher than that in the Japanese adult population (4.2%) [20]. While the present sample included more older respondents than these population-based studies, the prevalence of mental health problems, either moderate or severe, seems 1.5 times greater compared to that in the general population of Japan. The finding suggests that suvivors living in these affected areas had poorer mental health, as observed in many previous studies in Japan and other countries after natural disasters [2]–[5]. Further attention and appropriate long-term support are warranted for individuals with SMHPs and MMHPs.

The present study revealed factors associated with mental health among survivors. Women had significantly greater mental health problems than men. This finding is consistent with those of earlier studies assessing mental health problems among survivors of disasters [2], [10], [11]. It is also consistent with the findings of previous studies conducted in non-disaster settings [13], [14].

A survey of survivors of the 2008 Sichuan Earthquake has shown that older persons have a greater risk of mental health problems than younger persons have [27]. However, the present study was consistent with most other studies, which has indicated that prevalence of mental health problems is higher in younger or middle ages than older age [2], [8], [9]. In addition, our study has important implications for sex-specific intervention. Stratified analysis showed that there was a sex difference in age-specific patterns of prevalence of SMHPs. Compared with older age, younger and middle-aged persons were more likely to have SMHPs among men, while there was no such association between age and SMHPs among women. This may be attributable to the burden imposed on younger and middle-aged men. They are struggling to support their family after financial loss, and are in charge of reconstructing their community. Thompson et al have suggested that the middle-aged may be the most affected, compared to other age groups, because of their social and financial burden [28]. Moreover, men who suffered economic hardship had a greater risk for SMHPs than women did. Perceived obligation for their life may lead to an increase in mental health problems in men. Our results appear to underline the importance of economic support and employment, especially for men. Although the victims were able to find employment, some of these engagements were on a short-term or irregular basis. The creation of permanent jobs, in particular, has been emphasized. Measures to attract firms to invest in and establish plants in devastated areas are needed, as is support for victims seeking new employment. Our study demonstrated an association between frequency of relocation and mental health problems. A previous study has also suggested that relocation after a disaster increases psychological distress [12]. It is noteworthy that the OR for suffering from SMHPs among females with relocation experience was significantly higher than those with no such experience; no increase in OR was noted for males, though, after adjusting for covariates. In the immediate aftermath of the disaster, many survivors were forced to take shelter wherever they could. Those who experienced multiple relocations might have been subjected to cumulative stress in the disaster’s chaotic aftermath.

Consistent with previous findings [8], [27], a lack of social networks appeared to be significantly associated with mental health problems. Social resources, such as social networks, seem to be especially vulnerable to the impact of disasters. For example, disasters can remove significant supporters from victims’ networks through death [2]. Our study showed that nearly half of the respondents were classified as lacking social support. A study conducted before the disaster among residents of a neighboring community reported that approximately 20% lacked the support of a social network [29]. In the study areas, residents’ social networks were often smaller than before the disaster because many had lost their families, relatives, and friends. In addition, temporary housing communities in these areas tended to be relatively small, which could make it more difficult to recreate an environment or sense of community [23]. Survivors who have lost their social networks require appropriate care. Efforts to rebuild the community in the aftermath of the disaster persist until this day. Victims have begun moving from temporary housing to public housing built for them. However, the reconstruction of networks and communities that has begun to occur in temporary housing complexes may be seriously impacted by this move. Town-building efforts should therefore be accompanied by community conscious planning. The Great East Japan Earthquake and Tsunami hit the older aged communities along the shores of Tohoku. Our study revealed that older survivors were not necessarily more vulnerable to mental health problems than were younger survivors. Rather, the results suggested that survivors’ social circumstances, such as their lack of social networks as well as the presence of health complaints and severe economic hardships, were important risk factors. Considering the emergence of Japan’s super-aging society, our findings hold implications for research as well as practice with regard to the welfare of older populations in the wake of a disaster.

The present study had several limitations. First, the sample may not have been representative of the wider community. We collected questionnaires at the municipal health check-up sites. Even though >90% of the examinees participated in our study, those attending health check-ups tend to be more conscious about their health. In addition, those with serious mental health problems prefer to stay indoors, and may not have come for a health check-up. Therefore, it is possible that some of the high-risk populations were not represented in the data. Such self-selection biases may have led to the underestimation of mental health issues in our results. On the contrary, it cannot be denied that there is the possibility that mental health issues were over-reported. There may have been a higher concentration of respondents in our sample who had suffered directly as a result of the disaster. For example, the proportion of young adults aged <44_was relatively low in our study. Younger survivors who had some form of employment at the time were probably unable to join our study.

Second, although the K6 scale has been validated previously, it is not a clinical interview. Third, because this was a cross-sectional study, causal relationships could not be inferred from the data. For example, the associations between limited social networks and mental health problems are probably interactive. Social isolation may result in mental health problems, in turn could result in a limited social network. This issue should be examined in a follow-up study. Fourth, the correlations demonstrated in this study may not have adequately captured disaster specific patterns as they were not compared with those occurring in a non-disaster situation. We were not able to assess the damage caused by the disaster, such as the loss of property, family, and friends, or ask survivors if they lived alone in the present study. Due to the ethical issues involved in asking survivors still grieving the loss of a loved one to talk about their loss, we refrained from asking questions concerning their family members. In addition, we avoided using a voluminous questionnaire, given the burden it might pose on survivors. A challenge for future research is to combine data obtained on survivor health and welfare with another dataset on damage.

## Conclusions

Despite these limitations, to the best of our knowledge, this was the first epidemiological survey of mental health involving a large sample of residents living in Japan’s coastal areas after the 2011 Great East Japan Earthquake and Tsunami. In conclusion, our results indicated that mental health problems were prevalent among survivors living in these areas. Furthermore, females were more likely to suffer from mental health problems. Thus, there is a need to focus on their risk factors, including the presence of health complaints and severe economic hardships, as well as having insufficient social networks. For males, interventions focusing on economic support and those who are younger in age may be particularly useful in reducing mental health problems after the disaster.

## Supporting Information

Appendix S1 **Age- and sex-specific numbers of participants, acceptance rates, and proportions of total population in the study area.**
(XLSX)

Appendix S2 **Age- and sex-specific numbers of participants, acceptance rates, and proportions of total population in Yamada town.**
(XLSX)

Appendix S3 **Age- and sex-specific numbers of participants, acceptance rates, and proportions of total population in Otsuchi town.**
(XLSX)

Appendix S4 **Age- and sex-specific numbers of participants, acceptance rates, and proportions of total population in Rikuzentakata city.**
(XLSX)

## References

[1] Japanese National Police Agency (2012) Damage Situation and Police Countermeasures associated with 2011 Tohoku district - off the Pacific Ocean Earthquake. Available: http://www.npa.go.jp/archive/keibi/biki/higaijokyo_e.pdf. Accessed 2012 Nov 13.

[2] Norris FH, Friedman MJ, Watson PJ, Byrne CM, Diaz E, et al (2002) 60,000 disaster victims speak: Part I. An empirical review of the empirical literature, 1981–2001. Psychiatry 65: 207–239. 12405079 10.1521/psyc.65.3.207.20173

[3] Kessler RC, Galea S, Jones RT, Parker HA (2006) Mental illness and suicidality after Hurricane Katrina. Bull World Health Organ 84: 930–939. 17242828 10.2471/blt.06.033019 PMC1852424

[4] Frankenberg E, Friedman J, Gillespie T, Ingwersen N, Pynoos R, et al (2008) Mental health in Sumatra after the tsunami. Am J Public Health 98: 1671–1677. 18633091 10.2105/AJPH.2007.120915 PMC2509591

[5] Miguel-Tobal JJ, Cano-Vindel A, Gonzalez-Ordi H, Iruarrizaga I, Rudenstine S, et al (2006) PTSD and depression after the Madrid March 11 train bombings. J Trauma Stress 19: 69–80. 16568454 10.1002/jts.20091

[6] Shinfuku N (1999) To be a victim and a survivor of the great Hanshin-Awaji earthquake. Journal of Psychosomatic Research 46: 541–548. 10454170 10.1016/s0022-3999(98)00125-1

[7] Suzuki Y, Tsutsumi A, Fukasawa M, Honma H, Someya T, et al (2011) Prevalence of mental disorders and suicidal thoughts among community-dwelling elderly adults 3 years after the niigata-chuetsu earthquake. J Epidemiol 21: 144–150. 21325733 10.2188/jea.JE20100093 PMC3899506

[8] Oyama M, Nakamura K, Suda Y, Someya T (2012) Social network disruption as a major factor associated with psychological distress 3 years after the 2004 Niigata-Chuetsu earthquake in Japan. Environmental health and preventive medicine 17: 118–123. 21710149 10.1007/s12199-011-0225-y PMC3342634

[9] Seplaki CL, Goldman N, Weinstein M, Lin YH (2006) Before and after the 1999 Chi-Chi earthquake: traumatic events and depressive symptoms in an older population. Soc Sci Med 62: 3121–3132. 16423437 10.1016/j.socscimed.2005.11.059

[10] Johannesson KB, Lundin T, Frojd T, Hultman CM, Michel PO (2011) Tsunami-exposed Tourist Survivors Signs of Recovery in a 3-year Perspective. J Nerv Ment Dis 199: 162–169. 21346486 10.1097/NMD.0b013e31820c73d1

[11] Chen CH, Tan HK, Liao LR, Chen HH, Chan CC, et al (2007) Long-term psychological outcome of 1999 Taiwan earthquake survivors: a survey of a high-risk sample with property damage. Compr Psychiatry 48: 269–275. 17445522 10.1016/j.comppsych.2006.12.003

[12] Kilic C, Aydin I, Taskintuna N, Ozcurumez G, Kurt G, et al (2006) Predictors of psychological distress in survivors of the 1999 earthquakes in Turkey: effects of relocation after the disaster. Acta Psychiatr Scand 114: 194–202. 16889590 10.1111/j.1600-0447.2006.00786.x

[13] Inaba A, Thoits PA, Ueno K, Gove WR, Evenson RJ, et al (2005) Depression in the United States and Japan: gender, marital status, and SES patterns. Soc Sci Med 61: 2280–2292. 16115712 10.1016/j.socscimed.2005.07.014

[14] Kuriyama S, Nakaya N, Ohmori-Matsuda K, Shimazu T, Kikuchi N, et al (2009) Factors associated with psychological distress in a community-dwelling Japanese population: the Ohsaki Cohort 2006 Study. J Epidemiol 19: 294–302. 19749498 10.2188/jea.JE20080076 PMC3924098

[15] Artazcoz L, Benach J, Borrell C, Cortes I (2004) Unemployment and mental health: understanding the interactions among gender, family roles, and social class. Am J Public Health 94: 82–88. 14713703 10.2105/ajph.94.1.82 PMC1449831

[16] Takagi D, Kondo K, Kawachi I (2013) Social participation and mental health: moderating effects of gender, social role and rurality. BMC Public Health 13: 701. 23902596 10.1186/1471-2458-13-701 PMC3734140

[17] Ministry of Internal Affairs and Communications (2012) Eastern Japan pacific area data and damage report (in Japanese). Available: http://www.stat.go.jp/info/shinsai/. Accessed 2012 Oct 25.

[18] Kessler RC, Andrews G, Colpe LJ, Hiripi E, Mroczek DK, et al (2002) Short screening scales to monitor population prevalences and trends in non-specific psychological distress. Psychol Med 32: 959–976. 12214795 10.1017/s0033291702006074

[19] Furukawa TA, Kawakami N, Saitoh M, Ono Y, Nakane Y, et al (2008) The performance of the Japanese version of the K6 and K10 in the World Mental Health Survey Japan. Int J Methods Psychiatr Res 17: 152–158. 18763695 10.1002/mpr.257 PMC6878390

[20] Sakurai K, Nishi A, Kondo K, Yanagida K, Kawakami N (2011) Screening performance of K6/K10 and other screening instruments for mood and anxiety disorders in Japan. Psychiatry Clin Neurosci 65: 434–441. 21851452 10.1111/j.1440-1819.2011.02236.x

[21] Fukuda Y, Hiyoshi A (2012) Influences of income and employment on psychological distress and depression treatment in Japanese adults. Environ Health Prev Med 17: 10–17. 21431805 10.1007/s12199-011-0212-3 PMC3258311

[22] Kessler RC, Barker PR, Colpe LJ, Epstein JF, Gfroerer JC, et al (2003) Screening for serious mental illness in the general population. Arch Gen Psychiatry 60: 184–189. 12578436 10.1001/archpsyc.60.2.184

[23] World Health Organization Regional Office for the Western Pacific (2012) The Great East Japan Earthquake. Available: http://www.wpro.who.int/publications/docs/japan_earthquake.pdf. Accessed 2012 Oct 30.

[24] Lubben J, Blozik E, Gillmann G, Iliffe S, von Renteln Kruse W, et al (2006) Performance of an abbreviated version of the Lubben Social Network Scale among three European community-dwelling older adult populations. Gerontologist 46: 503–513. 16921004 10.1093/geront/46.4.503

[25] Kurimoto A, Awata S, Okubo T, Tsubota-Utsugi M, Asayama K, et al (2011) Reliability and validity of the Japanese version of the abbreviated Lubben Social Network Scale. Nippon Ronen Igakkai Zasshi 48: 149–157 (in Japanese).. 21778631 10.3143/geriatrics.48.149

[26] Sakurai K, Kawakami N, Yamaoka K, Ishikawa H, Hashimoto H (2010) The impact of subjective and objective social status on psychological distress among men and women in Japan. Soc Sci Med 70: 1832–1839. 20303205 10.1016/j.socscimed.2010.01.019

[27] Jia Z, Tian W, Liu W, Cao Y, Yan J, et al (2010) Are the elderly more vulnerable to psychological impact of natural disaster? A population-based survey of adult survivors of the 2008 Sichuan earthquake. BMC Public Health 10: 172. 20353554 10.1186/1471-2458-10-172 PMC2867995

[28] Thompson MP, Norris FH, Hanacek B (1993) Age differences in the psychological consequences of Hurricane Hugo. Psychol Aging 8: 606–616. 8292289 10.1037//0882-7974.8.4.606

